# Effect of anti-tuberculosis drugs on hematological profiles of tuberculosis patients attending at University of Gondar Hospital, Northwest Ethiopia

**DOI:** 10.1186/s12878-015-0037-1

**Published:** 2016-01-08

**Authors:** Eyuel Kassa, Bamlaku Enawgaw, Aschalew Gelaw, Baye Gelaw

**Affiliations:** Department of Hematology and Immunohematology, School of Biomedical and Laboratory Sciences, College of Medicine and Health Sciences (CMHS), University of Gondar (UOG), Gondar, Ethiopia; Department of Medical Microbiology, School of Biomedical and Laboratory Sciences, College of Medicine and Health Sciences, University of Gondar, Gondar, Ethiopia

**Keywords:** Tuberculosis, Haematological profile, Intensive phase, Anti-TB drugs

## Abstract

**Background:**

Tuberculosis (TB) treatment may present significant hematological disorder and some anti-TB drugs also have serious side effects. Although many other diseases may be reflected by the blood and its constituents, the abnormalities of red cells, white cells, platelets, and clotting factors are considered to be primary hematologic disorder as a result of tuberculosis treatment. The aim of this study was to determine hematological profiles of TB patients before and after intensive phase treatment.

**Objective:**

The aim of this study was to determine hematological profiles of TB patients before and after intensive phase treatment.

**Methods:**

Smear positive new TB patients were recruited successively and socio-demographic characteristics were collected using pre-tested questionnaire. About 5 ml of venous blood was collected from each patient and the hematological profiles were determined using Mindry BC 3000 plus automated hematology analyzer.

**Result:**

The hematological profiles of TB patients showed statistically significant difference in hematocrit (38.5 % versus 35.7 %), hemoglobin (12.7 g/lversus11.8 g/l) and platelet (268 × 10^3^/μlversus239 × 10^3^/μl) values of patients before initiation of treatment and after completion of the intensive phase of tuberculosis treatment, respectively (*P* < 0.05). The red cell distribution width (RDW) of treatment naïve TB patients was by far lower (17.6 ± 7.09 %) than the corresponding RDW (31.9 ± 5.19 %) of intensive phase treatment completed patients. Among TB patients that had high platelet distribution width (PDW) (*n* = 11) before initiation of TB treatment, 10 demonstrated lower PDW values after completion of the intensive phase. There was no significant difference on total white blood cell count among TB patients before and after completion of the 2 month treatment.

**Conclusion:**

The levels of hemoglobin, hematocrit and platelet count of the TB patients were significantly lowered after completion of the intensive phase of TB treatment. Significant variation of the RDW and PDW were also observed among treatment naïve and treatment completed patients. Hematological abnormalities resulted from TB treatment should be assessed continuously throughout the course of tuberculosis therapy.

## Background

Tuberculosis (TB) is a contagious disease that can affect almost any tissue and organs of the human body but mainly cause infection of the lungs [1]. Tuberculosis is a major public health problem throughout the world. About a third of the world’s population is estimated to be infected with tubercle bacilli and hence at risk of developing active TB disease. The burden of TB is highest in Africa, Asia, India and China together accounting for almost 40 % of the world’s TB cases [2]. About 2.4 million new TB cases and 540,000 TB-related deaths occur in sub-Saharan Africa annually [3]. Ethiopia is among the countries most heavily affected by TB [4]. The annual TB incidence of Ethiopia is estimated to be 341/100,000. Tuberculosis mortality rate is 73/100,000 and the prevalence of all forms of TB is estimated to be 546/100,000 [5].

TB is a curable disease although drug resistance is one of the major challenges in the treatment, prevention and control of the disease. However, the use of anti-TB drugs is not free from side effects. Allergic reactions, fever, rash, vasculitis, nausea, vomiting, hepatotoxicity, hepatocellular inflammation, peripheral neuropathy and others are some of the common side effects associated with TB treatment [6]. In addition, a wide range of hematological abnormalities has been reported as a result of administration of anti-TB drugs. The major hematological disorder due to anti-TB drug is aplastic anemia. Aplastic anemia is a hypo- regenerative bone marrow disorder characterized by a reduction in the amount of haemopoitic bone marrow and pancytopenia. It has been reported that pancytopenia due to aplastic anemia of moderate severity was observed in a patient while receiving anti-tuberculosis therapy, probably caused by an idiosyncratic reaction to streptomycin. Although the total leukocyte and platelet counts could be depressed after cessations of drugs, physicians rarely suspect anti-tuberculosis induced hematological complications which may have fatal consequences [7].

Hematological disorders arise through a variety of mechanisms and etiologies. Drug-induced hematological disorders can span almost the entire spectrum of hematology, affecting red cells, white cells, platelets, and the coagulation system. The wide spectrum of drug-induced hematologic syndromes is mediated by a variety of mechanisms, including immune effects, interactions with enzymatic pathways, and direct inhibition of hematopoiesis. Drug-induced syndromes include hemolytic anemia, red cell aplasia, sideroblastic anemia, megaloblastic anemia, polycythemia, aplastic anemia, leukocytosis and others. There are four possible relationships of tuberculosis to hematologic disease. These are the hematologic disease predisposes to tuberculosis reactivation. Drugs may cause idiosyncratic reactions, malabsorption, interference with iron metabolism, and hemolysis in patients with red blood cell enzyme deficiencies. Idiosyncratic reactions manifested by depression of any or all of the three cellular blood elements (white cells, red cells and platelets) together with the coagulation system may be caused by any of the anti-tuberculosis drugs [8, 9].

The magnitude of drug induced hematological abnormalities had been investigated in different parts of the world. For example, leucopenia as a result of rifampicin and isoniazid therapy was reported in Japan [10]. Anti-TB drug induced normocytic normochromic anemia was the most common abnormality observed in Malaysia [11] and a 74 % prevalence of anemia together with 26 % Leukocytosis, 24 % Thrombocytosis was reported in India [12]. In a study conducted in South Africa, a 15 % leucopenia, 23 % thrombocytopenia and 87 % lymphopenia was reported as a result of anti-TB drug treatment [13]. By another study conducted in Nigeria, anti-TB drug induced hematological abnormality were reported 93.6, 22.3, 45.2 and 4.8 % for anemia, leukocytosis, neutrophilia, and lymphopaenia, respectively [14]. Moreover, in Tanzania an 86 % anti-TB drug induced anemia was reported [15].

Different reports showed that altered hematopoiesis occurs in TB patients. Hematological changes associated with tuberculosis treatments have been investigated in many parts of the world. However, to the best of our knowledge, there is no comprehensive study assessed the hematological abnormalities among TB patients in Ethiopia in general and in Gondar in particular. Hence, this study was designed to determine the effect of anti-TB drugs on hematological profile among TB patients. In the present study, hematological findings amongTB patients before initiation and after completion of the intensive phase of tuberculosis treatment were assessed.

## Methods

### Study design, area and setting

A longitudinal prospective study was conducted from February to June 2014 at the University of Gondar (UoG) hospital, Northwest Ethiopia. The hospital is found in Gondar town. Gondar town is located in the North Gondar Zone of the Amhara Region, North of Lake Tana, Northwestern Ethiopia, and 748 km far from Addis Ababa. The town has a latitude and longitude of 12°36′N 37°28′E with an elevation of 2133 m above sea level. Based on figures from the Central Statistical Agency in 2008, Gondar has an estimated total population of 231,977. University of Gondar hospital is a referral hospital for Northwestern Ethiopia with more than 400 beds serving a population of about 5 million. The Hospital is one of the biggest tertiary level referral and teaching hospitals in the region. A large number of people from the surrounding zones and nearby regions visit the hospital both for inpatient and as an outpatient treatment.

### Populations

The source population was all TB suspected patients (both pulmonary and extra pulmonary) attending for health service at the University of Gondar Hospital. The study population were smear positive pulmonary tuberculosis patients and all confirmed extra-pulmonary TB patients who took all the intensive phase of treatment at the Directly Observed Treatment Short course (DOTS) clinic of University of Gondar hospital during the study period. In this study, TB patient less than 5 years of age and greater than 65 years of age, retreatment cases, patient with known organ impairment, malignant cases (cancer and chronic patients) and HIV patients were excluded from the study.

### Sample size determination and sampling technique

Time delimited consecutive sampling technique was employed to recruit the study subjects. All newly diagnosed TB patients who were confirmed for tuberculosis infection and seeking for anti-TB treatment at the University of Gondar Teaching Hospital DOTS clinic during the study period were included. By using the inclusion and exclusion criteria’s set for the study, a total of 168 new TB patients were recruited and involved in this study.

### Socio-demographic data

Socio-demographic characteristics such as age, sex, marital status, residence and clinical information such as WHO clinical stage, use of myelo-suppressive drugs, loss of appetite, weight loss, night sweats and fever were gathered using pre-tested and structured questionnaire. A trained clinical nurse and laboratory technologist working at the DOTS clinic of the University of Gondar hospital collected the socio-demographic characteristics of the TB patients.

### Sputum sample collection and microscopy

Sputum samples were collected using dry, clean, leak proof, translucent and screw-capped plastic containers with a capacity of 30 ml. Smears were prepared and air dried then stained with Ziehl-Neelsen (ZN) stain. Briefly, smears were genteelly heat fixed and each smear was flooded with carbolfuchsin solution. On each preparation, heat was applied until steaming and allowed to stand for 5 min. After washing with tap water, smear was decolorized with acid alcohol for 1 min and washed with tap water and counter stained with methylene blue for 30 s, washed and air dried. Slides were examined under 100× oil immersion objective with bright field illumination. Microscopy results were recorded following the WHO reporting methods as negative, actual number of acid-fast bacilli (AFB), 1+, 2+ and 3 + .

### Blood sample collection and determination of hematological parameters

About 5 ml of venous blood was collected aseptically by using EDTA tube from each of the selected study subjects. following collection, the EDTA tube was labeled with code number. The blood sample was collected from each study subject before initiation of anti-TB drugs and after completion of the 2 month intensive phase treatment. Hematologic profiles such as Red blood cell count, hemoglobin level, hematocrit (HCT), Red blood cell indices such as mean corpuscular volume (MCV), mean corpuscular haemoglobin (MCH), mean corpuscular haemoglobin concentration (MCHC), Red cell distribution width (RDW), total white blood cell count (WBC), WBC differential count, Platelet count, Mean Platelet Volume (MPV) and platelet cell distribution width (PDW) were determined using Mindray automated hematology analyzer following the manufacturers instruction and the Standard Operational Procedures (SOP) of the University of Gondar hospital laboratory.

### Quality control

The reliability of the study findings were guaranteed by implementing quality control (QC) measures throughout the whole process of the laboratory work. All materials, equipment and procedures were adequately controlled. Blood samples were collected free of hemolysis, clot, with sufficient quantity (5 ml) and correctly labeled with patients identification number. The performance of the hematology analyzer was check by running normal, low and high blood controls. During a condition where flags were found with automation hematological parameters, manual differential count was performed. Sputum samples with an AFB score of 1+ (*n* = 10) was used to assess the reliability of microscopic examinations. These samples were stained by Ziehl-Neelsen stain and examined by two laboratory technologists blinded from each other. The coefficient of variation (CV) was found 0.016 which shows that the result of the two laboratory technologists was 99.984 % consistent. The questionnaire was prepared in English, translated to local Amharic language and then translated back to English to check for consistency. Data on Socio-demographic and TB related medical history were collected by trained nurses under the supervision of the investigators.

### Data analysis

Data were checked, sorted, categorized and coded manually then transferred to SPSS version 20 statistical packages for analysis. Frequencies and cross tabulations were used to summarize descriptive statistics. Paired *t* test was used in the analysis to compare the hematologic values before initiation and after completion of the intensive phase of tuberculosis treatment. A *P* values less than 0.05 were considered statistically significant.

### Ethical consideration

Ethical approval was obtained from an ethical review committee organized by the School of Biomedical and Laboratory Sciences which was mandated by the College of Medicine and health Sciences, University of Gondar. A permission and support letter was also obtained from University of Gondar hospital and both verbal and written consent was obtained from each participant. All the consent procedures were approved by the ethical approval committee of the School of Biomedical and Laboratory Sciences. In the case of children, both verbal and written consent were taken from the guardian that was also approved by the ethical review committee. The purpose and importance of the study was explained to each study participants. To ensure confidentiality of participants information, anonymous typing was applied where by the name of the participant and any identifier of participants were not written on the questionnaire, and during the interview to keep the privacy, they were interviewed alone. The laboratory findings of each study participant were communicated with the responsible clinician assigned at TB clinic. Above all the data was collected after full written consent was obtained from each study participates.

## Results

### Characteristics of the study participants

A total of 168 HIV negative new TB patients were included in this study. Ninety six (56.5 %) of the TB patients were males and the other 73 (43.5 %) were females. The mean age of the study participants was 34.8 ± 15.3 years ranging from 5 to 65 years. Sixty-two (36.9 %) TB patient were within the age group 5–25, 62 (36.9 %) within the age group 26–44 and the other 44 (26.2 %) within 45–65 years of age. Forty-seven percent (*n* = 79) were urban dwellers and the other 53 % (*n* = 89) were living in rural areas. The clinical and laboratory data of the TB patients showed that 87 (51.8 %) and 81 (48.2 %) were pulmonary and extra pulmonary cases.

### Association between hematological parameters with the age and sex of the TB patients before and after completion of the intensive phase treatment

Tuberculosis confirmed patients were investigated to assess their hematological profile prior initiation of anti-TB treatment and after completion of the intensive phase treatment. The hematological profiles assessed were red blood cell count, hemoglobin concentration, hematocrit, red cell indices, total white blood cell count, white blood cell differential count, red blood cell distribution width, platelet distribution width, platelet count and mixed cells (eosinophile, monocyte and basophile) count. Analysis of the demographic characteristics of the TB patients such as age and sex versus hematological parameters demonstrated that there was significant difference on Hgb and Hct concentrations (*P* < 0.001) before initiation and after completitions of the intensive phase treatment. In addition, a significant difference on RBC among patients with the age group 45–65 years was observed (*P* = 0.030). The RDW-CV parameter of female patients and the PLT count of male patients were also significantly different (*P* = 0.002 and 0.031 respectively) (Table 1). However, there was no significant difference in WBC parameters and PDW versus the age and sex of the TB patients.

**Table 1:** Hematological parameters that demonstrated significant difference based on the age and sex of TB patients before initiation of anti-TB drugs compared with after completion of the intensive phase of treatment

			Before treatment	After treatment	*P*-value
RBC × 10^6^/ μl	Sex	Male	4.27 ± 0.92	4.43 ± 0.92	0.235
		Female	4.24 ± 0.72	4.42 ± 0.68	0.143
	Age	5 – 25	4.26 ± 0.77	4.42 ± 0.89	0.320
		26 – 44	4.37 ± 0.75	4.33 ± 0.68	0.747
		45 – 65	4.09 ± 1.01	4.58 ± 0.92	0.030*
Hgb (g/dl)	Sex	Male	12.7 ± 2.1	11.8 ± 1.5	0.000*
		Female	12.8 ± 2.1	11.8 ± 1.9	0.000*
	Age	5 – 25	12.9 ± 2	11.8 ± 1.8	0.000*
		26 – 44	12.9 ± 1.8	12 ± 1.4	0.001*
		45 – 65	12.4 ± 2.5	11.5 ± 1.8	0.000*
HCT (%)	Sex	Male	38.6 ± 6	35.8 ± 4.4	0.000*
		Female	38.9 ± 5.5	35.7 ± 5.4	0.000*
	Age	5 – 25	39.1 ± 5.7	35.7 ± 5.5	0.000*
		26 – 44	38.9 ± 4.9	36.6 ± 4	0.001*
		45 – 65	37.9 ± 6.8	34.7 ± 5	0.000*
MCV (fl)	Sex	Male	88.4 ± 6.9	89.4 ± 9.1	0.036*
		Female	89.4 ± 6.7	90.9 ± 5.1	0.143
	Age	5 – 25	89.4 ± 7.3	90.2 ± 5.1	0.486
		26 – 44	88.3 ± 5.8	91.2 ± 5.8	0.013*
		45 – 65	88.7 ± 7.6	90.4 ± 5.2	0.241
MCH (pg)	Sex	Male	28.9 ± 3.3	29.6 ± 2.3	0.143
		Female	29.2 ± 2.6	30.1 ± 2	0.040*
	Age	5 – 25	29.3 ± 3	29.7 ± 2.1	0.427
		26 – 44	29.1 ± 3.1	29.7 ± 2.5	0.253
		45 – 65	28.5 ± 3	30 ± 2	0.012*
MCHC (%)	Sex	Male	32.7 ± 2	32.7 ± 1.3	0.878
		Female	32.6 ± 1.4	33.1 ± 1.1	0.049*
	Age	5 – 25	32.6 ± 1.6	32.9 ± 1.3	0.357
		26 – 44	33 ± 1.9	32.7 ± 1.3	0.390
		45 – 65	32.2 ± 1.6	33.1 ± 1	0.003*
RDW CV (%)	Sex	Male	14.4 ± 1.4	14.2 ± 1.2	0.311
		Female	14.5 ± 1.7	13.9 ± 1.2	0.02*
	Age	5 – 25	14.4 ± 1.4	14.1 ± 1.3	0.143
		26 – 44	14.3 ± 1.4	14.1 ± 1.2	0.477
		45 – 65	14.6 ± 1.8	14 ± 1.1	0.059
PLT × 10^3^/μl	Sex	Male	267 ± 106	232 ± 109	0.031*
		Female	270 ± 101	250 ± 86	0.169
	Age	5 – 25	258 ± 105	250 ± 116	0.697
		26 – 44	274 ± 101	246 ± 72	0.085
		45 – 65	275 ± 105	216 ± 108	0.006*

* = significant association

### Assessment of anemia

The magnitude of anemia among TB patients before initiation and after completion of the intensive phase of treatment was assessed using red blood cell count (RBC), hemoglobin (Hgb) and hematocrit (Hct) values. The average RBC of the TB patients before initiation of anti-TB treatment was relatively similar to that of RBC after completion of the intensive phase of tuberculosis treatment (4.25 × 10^3^ ± 0.83 versus 4.42 × 10^3^ ± 0.824, respectively) (Table 2). Many of the TB patients that had normal RBC before initiation of tuberculosis treatment (*n* = 72) demonstrated also normal RBC after completion of the intensive phase (*n* = 69) of treatment. However, 54 % of the TB patients had lower RBC before initiation and after completion of the intensive phase of treatment. Nevertheless, data showed no statistically significant difference on the RBC of TB patients before initiation and after completion of the intensive phase of tuberculosis treatment. The average Hgb concentration of the TB patients before initiation of tuberculosis treatment was slightly higher (12.7 ± 2.09 g/dl) than the corresponding Hgb concentration determined after completion of the intensive phase of treatment (11.8 ± 1.68 g/dl). Similarly, the average packed cell volume (Hct) was also relatively higher before initiation of treatment (38.5 ± 2.42 %) than the corresponding concentration after completion of the 2 month treatment (35.7 ± 2.31 %). In this study, 55 % of the TB patients (*n* = 92) had low Hgb concentration and the Hct values of 86 TB patients (51 %) was found low before initiation of anti-tuberculosis treatment. The proportion of TB patients with low Hgb and Hct concentration was by far increased (72 %) after completion of the intensive phase of TB treatment. Among the TB patients that had higher Hct concentration (*n* = 13) before initiation of anti-TB treatment, only 4 resulted similarly higher concentration. On the other hand, among 69 and 76 patients that had normal Hct and Hgb concentration before initiation of anti-TB treatment, only 42 and 47 demonstrated normal concentrations respectively after completion of the intensive phase treatment (Table 3). Moreover, data showed a significant decreased level of Hgb concentration and Hct value after completion of the 2 month tuberculosis treatment compared to the pre-treatment level (*P* = 0.00).

**Table 2:** Hematological profiles of tuberculosis patients before and after initiation of anti-TB treatment at University of Gondar Teaching hospital, 2014

Variable	Before treatmentof TB Mean (SD)	After treatmentof TB Mean (SD)	P-Value
RBC/μl	4.25 × 10^6^ (0.83)	4.42 × 10^6^ (0.824)	0.072
WBC/ μl	7.5 × 10^3^ (3.76)	7.08 × 10^3^ (3.27)	0.224
Hgb (g/dl)	12.7 (2.09)	11.8 (1.68)	0.000*
HCT (%)	38.5 (2.42)	35.7 (2.31)	0.000*
MCV (fl)	88.8 (6.8)	89.7 (8.4)	0.268
MCH (Pg)	30.13 (5.0)	29.03 (3.0)	0.018*
MCHC (g/dl)	30.13 (2.91)	29.43 (7.1)	0.000*
RDW-CV	17.6 (7.09)	31.9 (5.19)	0.000*
PLT/ μl	268 × 10^3^ (103.2)	239.93 (99.8)	0.01*
PDW	17.01 (4.79)	15.7 (0.61)	0.001*
Lymphocyte	1.96 × 10^3^ (0.94)	2.08 × 10^3^ (1.36)	0.375
Mixed cell	0.74 × 10^3^ (0.422)	0.69 × 10^3^ (0.425)	0.272
Neutrophil	4.37 × 10^3^ (2.42)	4.08 × 10^3^ (2.31)	0.230

*RBC* red blood cell, *WBC* White blood cell, *HCT* Haematocrit, *HGB* Haemoglobin, *MCV* mean corpuscular volume, *MCH* mean haemoglobin, *MCHC* mean haemoglobin concentration, *RDW-CV* red cell distribution width, *PLT* platelet, *PDW* Platelet distribution width, *fl* femto litter (10^−15^L), *pg* pico-gram (10^−12^g), * significant association

**Table 3:** The proportion of TB patients with low, normal and high hematological profile before initiation and after completion of the intensive phase tuberculosis treatment at University of Gondar hospital, 2014

Parameters	Category	Before TB treatment	After TB treatment	Reference Intervals	*P*-Value
RBC	Low	91 (54.2)	92 (54.8)		0.92
	Normal	72 (42.9)	69 (41.1)	M = 4.5-6.2 × 10^6^/ μl	
				F = 4.0 -5.5 × 10^6^/μl	
	High	5 (3)	7 (4.2)		
WBC	Low	24 (14.3)	31 (18.5)		0.262
	Normal	115 (68.5)	113 (67.3)	4.0 – 10.0 × 10^3^/μl	
	High	29 (17.3)	24 (14.3)		
HCT	Low	86 (51.2)	122 (72.6)		0.000*
	Normal	69 (41.1)	42 (25.0)	M = 40 -52 %	
				F =36 – 47 %	
	High	13 (7.7)	4 (2.4)		
HGB	Low	92 (54.8)	121 (72)		0.000*
	Normal	76 (45.2)	47 (28)	M = 13 – 18 g/dl	
				F = 12–16 g/dl	
	High	0 (0)	0 (0)		
MCV	Low	13 (7.7)	10 (6)		0.589
	normal	129 (76.8)	130 (77.4)	80 – 100 fl	
	high	26 (15.5)	28 (16.7)		
MCH	low	37 (22)	18 (10.7)		0.000*
	Normal	96 (57.1)	105 (62.5)	27 – 32 pg	
	High	35 (20.8)	45 (26.8)		
MCHC	low	75 (44.6)	163 (98.2)		0.000*
	normal	90 (53.6)	3 (1.8)	31.5 – 36 g/dl	
RDW_CV	Normal	118 (70.2)	9 (5.4)	11.5 - 14.5 %	0.000*
	high	50 (29.8)	159 (94.6)		
PLT	low	22 (13.1)	25 (14.9)		0.01*
	normal	113 (67.3)	128 (76.2)	150 - 450 × 10^3^/μl	
	High	33 (19.6)	15 (8.9)		
PDW	Normal	157 (93.5)	167 (99.4)	10 -17 %	0.000*
	high	11 (6.5)	1 (0.6)		
Lymphocyte	low	4 (2.4)	6 (3.6)		1.000
	Normal	160 (95.2)	156 (92.9)	1.5 - 4.5 × 10^3^/μl	
	high	4 (2.4)	6 (3.6)		
Mixed cell	Normal	160 (95.2)	165 (98.2)	0.26 -1.3 × 10^3^/μl	0.960
	high	8 (4.8)	3 (1.8)		
Neutrophil	Low	13 (7.7)	24 (14.3)		0.233
	Normal	136 (81)	124 (73.8)	2.0 – 7.0 × 10^3^/μl	
	High	19 (11.3)	20 (11.9)		

* = significant association. Hematological parameter values less than and greater than the reference intervals are categorized as low and high respectively

### Leukocytosis versus leukopenia

The average total WBC of TB patients before treatment (7.5 × 10^3^ ± 3.76) was slightly lower than the corresponding average WBC after completion of the 2 month treatment (7.08 × 10^3^ ± 3.27). The total WBC of 24 TB patients (14.3 %) enumerated before initiation of TB treatment was low but that of 115 (68.5 %) and 29 (17.3) patients were normal and high, respectively. The proportion of tuberculosis patients with low WBC was slightly raised 18.5 % (*n* = 31) when examined after completion of the intensive phase of treatment. However, there was no statistically significant difference on total WBC among TB patients before initiation and after completion of the 2 month tuberculosis treatment. The lymphocyte, neutrophil and mixed cell (eosinophil, monocyte and basophil) differential WBC count of the TB patients showed no statistically significant difference among TB patients before and after initiation of tuberculosis treatment. However, the proportion of TB patients with high neutrophil count was raised from 7.7 % (*n* = 13) of treatment naïve phase to 14.3 % (*n* = 24) after completion of the 2 month tuberculosis treatment.

### Association of tuberculosis treatment with platelet count

The mean thrombocyte count among TB patients before administration of anti-TB drugs was 268 × 10^3^ ± 103.2. The corresponding platelet counts among TB patients after completion of the 2 month treatment was slightly lower (239 × 10^3^ ± 99.8). However, data showed no statistically significant difference on platelet count among TB patients before initiation and after completion of the intensive phase tuberculosis treatment. The proportion of TB patients with low platelet count was slightly increased after completion of tuberculosis treatment (*n* = 25; 14.9 %) compared to the corresponding platelet count among tuberculosis treatment naïve patients (*n* = 22; 13.1 %). On the other hand, significantly reduced proportion of TB patients with high platelet count was observed (8.9 %; *n* = 15) after completion of the intensive phase of TB treatment compared to the proportion of TB patients with high platelet count (19.6 %; *n* = 33) among treatment naïve TB patients. Moreover, data showed significant difference in the proportion of TB patients with both low and high platelet count after completion of the 2 month treatment compared with treatment naïve patients (*P* = 0.01).

### Red blood cell indices during tuberculosis treatment

The average MCV of the TB patients before initiation of tuberculosis treatment and after completion of the 2 month treatment was nearly similar (88.8 ± 6.8 versus 89.7 ± 8.4, respectively). A slightly reduced MCH (29.03 ± 3.0) was observed after completion of the intensive phase tuberculosis treatment compared with the average MCH before initiation of treatment (30.13 ± 5.0). Similarly, the MCHC was also slightly reduced after completion of the 2 month treatment compared with before initiation of treatment (29.43 ± 7.1 versus 30.13 ± 2.91, respectively). The difference in MCH and MCHC among TB treatment naïve and TB patients completed the intensive phase was found statistically significant (*P* ≤ 0.018). In this study, 7.7 % (*n* = 13) of the TB patients had low MCV before initiation of anti-TB treatment. The proportion of TB patients with low MCV was slightly reduced (6 %; *n* = 10) after completion of the intensive phase treatment. The majority of the TB patients (57.1 %; *n* = 96) had normal MCH before initiation of treatment and the proportion of TB patients with normal MCH risen to 108 (62.5 %) after completion of the intensive phase treatment. Forty-five percent (*n* = 75) of the TB patients had low MCHC before initiation of treatment. The proportion of TB patients that had low MCHC was significantly raised (98.2 %; *n* = 163) after completion of the intensive phase treatment. The proportion of TB patients with normal MCHC before initiation of TB treatment was 53.6 % (*n* = 90). However, only 1.8 % (*n* = 3) of the TB patients that had normal MCHC before initiation of tuberculosis treatment had also normal MCHC after completion of the intensive phase treatment.

### Red blood cell and Platelet distribution width during tuberculosis treatment

The mean red blood cell distribution width (RDW) among treatment naïve TB patients (17.6 ± 7.09 %) was by far lower than the corresponding RDW (31.9 ± 5.19 fl) of TB patients determined after completion of the intensive phase of treatment. This difference was also statistically significant (*P* = 0.00). Contrary to RDW, the mean platelet distribution width (PDW) among treatment naïve TB patients (17.01 ± 4.79 %) was higher than that of TB patients completed the intensive phase of treatment (15.7 ± 0.61 %). Moreover, data also showed statistically significant difference on PDW among treatment naïve TB patients compared with TB patients completed the intensive phase of treatment (*P* = 0.00) (Table 2).

In this study, 70.2 % (*n* = 118) of TB patients had normal RDW before initiation of anti-TB treatment. However, the proportion of TB patients that had normal RDW was by far reduced to 5.4 % (*n* = 9) after completion of the intensive phase treatment. On the other hand, the proportion of TB patients with high RDW which was 29.8 % (*n* = 50) was raised to 94.6 % (*n* = 159) after completion of the intensive phase of tuberculosis treatment. Moreover, data showed statistically significant difference on the proportion of TB patients with high/low RDW before initiation and after completion of the intensive phase of tuberculosis treatment (*P* = 0.00) (Table 3).

The proportion of TB patients that had normal PDW (93.5 %; *n* = 157) before initiation of treatment was relatively similar to the corresponding proportion of TB patients that had normal PDW (99.4 %; *n* = 167) after completion of the 2 month treatment. Eleven TB patients (6.5 %) had high PDW before initiation of tuberculosis treatment but only 1 patient showed high PDW after completion of the intensive phase of treatment and the difference was statistically significant (*P* = 0.00).

## Discussion

Tuberculosis exerts incredible varieties of hematologic effects and hematologic abnormality is a common finding among TB patients. These abnormalities involves both cell lines and plasma components. Anti-tuberculosis therapy has its own spectrum of hematologic toxicity and blood cell abnormalities [16]. The current study compared the hematologic profile of HIV negative TB patients before initiation of treatment and after completion of the intensive phase of tuberculosis treatment.

In the current study, the average RBC of the TB patients before initiation of anti-TB treatment was relatively similar to that of RBC after completion of the intensive phase of tuberculosis treatment. The average RBC of the TB patients were 4.25 × 10^6^ ± 0.83 versus 4.42 × 10^6^ ± 0.824 before treatment and after completion of the intensive phase treatment, respectively, which is nearly similar to the lower limits of the reference values for male and female adults (4.7 and 4.2 million cells per microliter (cells/μL), respectively). Previously Tsegay et al. [17] reported reference range of erythrocyte counts, 5.1 × 10^12^/l (males) and 4.5 × 10^12^/l (females) for healthy adult Ethiopians.

The hemoglobin concentration and packed cell volume were slightly higher among treatment naïve TB patients compared with those received anti-TB treatment for 2 months. However, the proportion of TB patients with low hemoglobin and hematocrit concentration was significantly increased (72 %) after completion of the intensive phase of TB treatment. All chronic infections including TB can cause anemia [18]. Various pathogenesis have been suggested in TB-associated anemia, but most studies have been shown suppression of erythropoiesis by inflammatory mediators as a cause of anemia [19]. Nutritional deficiency and malabsorption syndrome can deepen the severity of anemia [20]. The possible mechanisms for the development of anemia during tuberculosis infection may be due to nutritional insufficiency, impaired iron utilization, mala-absorption, bone marrow granuloma and shortened duration of RBC survival [21]. Weiss [18], Means [22] and Nemeth et al. [23] explain the mechanism behind the occurrence of anemia in pulmonary tuberculosis patients saying that the invasion of bacteria leads to activation of T-lymphocyte and macrophages, which induce the production of the cytokines like interferon gamma (IFN-γ), tumor necrosis factor alpha (TNF-α), Interlukin-1 (IL-1) and interlukin-6 (IL-6) which with their products will cause diversion of iron into iron stores in the reticulo-endothelial system resulting in decreased iron concentrations in the plasma thus limiting its availability to red cells for hemoglobin synthesis, inhibition of erythroid progenitor cell proliferation and in appropriate production and activity of erythropoietin which may lead to anemia and suboptimal response of the bone marrow to anemia, respectively. The high proportion of TB patients with reduced hemoglobin and hematocrit concentration after completion of the intensive phase of tuberculosis treatment may suggest drug induced anemia among TB patients. Drugs can induce variety of hematological disorders affecting RBCs, WBCs and plateltes. Drug induced syndromes includes hemolytic anemia, methemoglobinemia, red cell aplasia, sideroblastic anemia, megaloblastic anemia, polycythemia and aplastic anemia [8].

The total WBC count of TB patients before initiation of anti-TB drugs was relatively similar to that of total WBC count after completion of the intensive phase of treatment. However, the proportion of TB patients with low total WBC count was raised from 14.3 % before initiation of treatment to 18.5 % after completion of the intensive phase treatment. On the other hand, the proportion of TB Patients with high neutrophil differential count was increased from 7.7 % before treatment to 14.3 % after completion of the intensive phase of treatment. Nevertheless, there was no leukocytosis neither before initiation of treatment nor after completion of the 2 month treatment. In a study designed to assess the hematological abnormalities of TB patients, Singh et al. [24] reported a 25 % leukopenia and a 22 % neutropenia among TB patients. Drug induced neutropenia was also reported in association with various analgesics, psychotropic’s, anti-convulsions, anti-thyroid drugs, anti-histaminics, anti-rheumatics, gastro-intestinal drugs, antimicrobials and cardiovascular drugs [25].

The result of the current study showed that platelet count was another important hematological profile that showed significant difference when the count before initiation of tuberculosis treatment was compared to that of platelet count after completion of the 2 month tuberculosis treatment (*P* = 0.010). Almost half of the TB patients that had high platelet count before treatment showed decreased platelet count after completion of the intensive phase. This finding is supported by a report from India and was suggested that decrease in platelet count could possibly be due to the effect of anti-TB drugs and immune destruction of platelets [26]. Classical causes of drug-induced thrombocytopenia are the quinine and quinine-like drugs [27]. The thrombocytopenia induced by these drugs is caused by antibody that is non-reactive in the absence of drug, but binds to epitopes on platelet membrane, glycoproteins IIb/IIIa or Ib/IX, when the sensitizing drug is present. Vancomycin can also be associated with marked thrombocytopenia and demonstrable drug-dependent antibodies in the serum [28]. Other drugs associated with thrombocytopenia include antimicrobials (sulfanomides, rifampin, linezolid), anti-inflammatory drugs, anti-neoplastics, anti-depressants, benzodiazepines, anti-convulsants (carbamazepine, phenytoin, valproic acid) as well as cardiac and anti-hypertensive drugs [29, 30].

In the current study, the MCV values before and after treatments were not significantly changed. However, the differences in the MCH and MCHC values were significantly different when the values were compared before and after completion of the intensive phase of TB treatment. Previous reports showed that the red cell indices in the untreated male pulmonary TB patients showed lower values as compared to normal males. In addition, it was also found that the MCH and MCHC among male pulmonary TB patients were significantly lower when compared with normal males [31, 32]. Different study reports showed that after anti-tuberculosis treatment with streptomycin, rifampicin and isoniazid, the RBC indices were affected and reached closer to normal values. Moreover, RBC morphology in pulmonary TB patients was found to be mainly normocytic normochromic type and during medication, the blood film showed normochromic pictures [31, 33, 34] which suggests the effects of tuberculosis treatment on the restoration of RBC morphology.

The mean RDW of TB patients determined after completion of the intensive phase of treatment (31.9 ± 5.19 %) was significantly different from the corresponding RDW of treatment naïve TB patients (17.6 ± 7.09 %). Opposite to the RDW, the PDW among treatment naïve TB patients (17.01 ± 4.79 %) was higher than the PDW of TB patients measured after completing the intensive phase of treatment (15.7 ± 0.61 %). Moreover, TB patients that had high PDW before treatment (*n* = 10) showed reduced PDW after completion of the 2 month anti-TB treatment. Red cell distribution width and PDW measure the variation of RBC and platelete volume. The normal reference range of RDW-CV in adult humans was reported 11.5 to 14.5 % [35] while that of PDW 9–16.56 % for men and 8–13.3 % for women [36]. Nowadays, RDW is used to diagnose anemia. Domingo et al. [37] reported greater decrease in hemoglobin concentration in patients with higher values for RDW. Red cell distribution width is often used to differentiate anemia of mixed causes from anemia of a single cause. There are reports that documented deficiency of vitamin B12, or folate produce macrocytic anemia (large cell anemia) in which the RDW is elevated in roughly two-thirds of all cases [38]. However, varied size distribution of RBC is the hallmark of iron deficiency anemia, and as such shows increased RDW in virtually all cases [39]. Long term exposure to anti-tuberculosis medication increases the risk of adverse drug reactions and toxicity. Isoniazid and rifampicin may directly cause hemolytic anemia, as can pyrazinamide cause sidroblastic anemia [40]. Others have suggested that anemia is seen as part of the clinical manifestation of tuberculosis and as a consequence of a chronic disease. In general, tuberculosis patients have a higher predisposition to develop gastro-intestinal absorption problems, consequently leading to anemia [20] and elevated RDW is associated with anemia. At a condition when there is microcytic RBCs produced prior to treatment and normocytic RBCs produced after completion of treatment in the circulation leads to high size variation (increase RDW value). Lee et al. [20] showed that anemia is a common hematological abnormality in patients and Baynes et al. [19] states that RDW values in chronic inflammatory disorder like tuberculosis similar with the RDW values occurring in iron-deficiency anemia which is in line with the current study.

Platelet distribution width directly measures the variability in platelet size and has been used to differentiate disorders of platelets such as essential thrombocythemia from reactive thrombocytosis [41]. In the present study, PDW of TB patients was significantly reduced after taking anti-TB drugs for 2 months. Previously, Tozkoparan et al. [42] reported that there were significantly higher PDW values (40 ± 23.5) among TB patients which decreased significantly with anti-tuberculosis therapy. Thrombocytopenia is a serious side effect that potentially caused by anti-TB drugs which occurs mostly due to rifampicin (RIF) [43]. The main mechanisms of thrombocytopenia are decreased production or increased destruction of platelets. The drug binds non-covalently to membrane glycol-proteins to produce compound epitopes or induce conformational changes which antibodies are specific. In addition, RIF-dependant antibodies attach to thrombocytes and cause increased destruction [44]. However, the exact mechanism of INH-induced thrombocythemia is not known.

The result of this study showed that the Hgb concentration, HCT, PLT and PDW values were reduced after completion of the intensive phase of tuberculosis treatment. This is due to the fact that iron is critical for *Mycobacterium tuberculosis* growth in macrophages in turn causes iron deficiency anemia. After the intensive phase treatment, there will be sufficient iron in the body and starts production of normal erythrocytes. This study finding is supported by Tozkoparan et al. [42] that indicates significantly lower PDW values with anti-tuberculosis therapy. Also another study by Sahin et al. [45] indicates that reactive thrombocytosis and PDW develop frequently in PTB and there is a relation with acute phase reactants, which is the inflammatory response and decreased after treatment.

## Conclusion

The levels of hemoglobin, hematocrit and platelet count of the TB patients were significantly lowered after completion of the intensive phase of TB treatment compared with the corresponding values before initiation of anti-TB treatment. The RDW and PDW also showed significant variation before initiation and after completion of the 2 month tuberculosis treatment. The varied hematological abnormalities observed in TB patients after intensive phase tuberculosis therapy suggests the need for continuous monitoring and evaluation of TB patients for adverse hematological abnormalities during tuberculosis treatment. Anemia was found to be one of the most common hematological abnormalities among patients taking anti-tuberculosis treatment. Thus anemia and thrombocytopenia should be regularly checked during tuberculosis treatment.

### Limitation of the study

The limitation of this study was the relatively small sample size which is the result of the nature of the study as longitudinal study are time consuming and costly. We were also unable to determine the effect of anti-TB drugs after completion of the 6 month treatment.
